# Membrane Potential-Dependent Modulation of Recurrent Inhibition in Rat Neocortex

**DOI:** 10.1371/journal.pbio.1001032

**Published:** 2011-03-22

**Authors:** Jie Zhu, Man Jiang, Mingpo Yang, Han Hou, Yousheng Shu

**Affiliations:** Institute of Neuroscience, State Key Laboratory of Neuroscience, Shanghai Institutes for Biological Sciences, Chinese Academy of Sciences, Shanghai, P. R. China; European Brain Research Institute, Italy

## Abstract

Dynamic balance of excitation and inhibition is crucial for network stability and cortical processing, but it is unclear how this balance is achieved at different membrane potentials (*V*_m_) of cortical neurons, as found during persistent activity or slow *V*_m_ oscillation. Here we report that a *V*_m_-dependent modulation of recurrent inhibition between pyramidal cells (PCs) contributes to the excitation-inhibition balance. Whole-cell recording from paired layer-5 PCs in rat somatosensory cortical slices revealed that both the slow and the fast disynaptic IPSPs, presumably mediated by low-threshold spiking and fast spiking interneurons, respectively, were modulated by changes in presynaptic *V*_m_. Somatic depolarization (>5 mV) of the presynaptic PC substantially increased the amplitude and shortened the onset latency of the slow disynaptic IPSPs in neighboring PCs, leading to a narrowed time window for EPSP integration. A similar increase in the amplitude of the fast disynaptic IPSPs in response to presynaptic depolarization was also observed. Further paired recording from PCs and interneurons revealed that PC depolarization increases EPSP amplitude and thus elevates interneuronal firing and inhibition of neighboring PCs, a reflection of the analog mode of excitatory synaptic transmission between PCs and interneurons. Together, these results revealed an immediate *V*_m_-dependent modulation of cortical inhibition, a key strategy through which the cortex dynamically maintains the balance of excitation and inhibition at different states of cortical activity.

## Author Summary

Proper functioning of the neocortex requires a balance between excitation and inhibition. This balance can be achieved through the operation of cortical microcircuits interweaved by excitatory and inhibitory neurons. Since the membrane potentials (*V*_m_) of cortical neurons fluctuate at different levels during cortical activities, it is important to know how the balance of excitation and inhibition is dynamically maintained at different *V*_m_. Recurrent inhibition between excitatory pyramidal cells is mediated by two distinct types of inhibitory interneurons. Here, we show that the amount of recurrent inhibition depends on the *V*_m_ levels of presynaptic pyramidal cells. Modest depolarization of a pyramidal cell substantially increases, and sometimes turns on, disynaptic inhibition on its neighboring pyramidal cells. We find that this effect is due to an increase in the strength of synaptic connections from the pyramidal cell to inhibitory interneurons and a consequent elevation of interneuronal firing. The depolarization-induced increase in synaptic strength from the pyramidal cell therefore reflects “analog-mode” signaling in cortical excitatory synapses. We thus reveal a profound impact of analog-mode signaling on the operation of cortical microcircuits and provide a new mechanism for dynamic control of the balance of cortical excitation and inhibition.

## Introduction

The excitatory and inhibitory inputs received by cortical neurons are normally under dynamic balance during cortical functions [1]–[7]. The interaction among these inputs, together with intrinsic membrane properties of cortical neurons, often results in shifts of membrane potential (*V*_m_) between different states [8]–[11], which could regulate neuronal responsiveness to synaptic and sensory inputs [1],[12]–[17]. However, it is unclear how the balance of excitation and inhibition is achieved when cortical neurons are at different *V*_m_ levels. During global membrane oscillations involving a large number of cortical neurons, excitation and inhibition may be proportionally altered by the *V*_m_ shift, but the underlying mechanisms remain unknown, in view of diversity of connectivity and functions of local inhibitory interneurons [18],[19]. In the case of persistent activities associated with some behaviorally relevant conditions, e.g., during working memory, a subpopulation of neurons undergoes changes in the *V*_m_ [20]–[24]. Microcircuits involving these active neurons also require dynamic control of their excitation-inhibition balance. In this study, we investigated how *V*_m_ changes of a cortical neuron may modulate the efficacy of recurrent inhibition within the microcircuit.

It has been shown recently that cortical excitatory neurons communicate not only through the generation of all-or-none action potentials (APs, digital mode) but also through a presynaptic *V*_m_-dependent modulation of transmitter release (analog mode) [25],[26]. It remains unknown to what extent the analog-mode communication influences the operation of local circuitry and has a functional role in the cortex. Considering that interneurons within the microcircuit are driven by excitatory neurons, leading to recurrent inhibition, we hypothesized that the amount of recurrent inhibition might be subjected to modulation in a manner that depends on the level of depolarization of the excitatory neuron. The *V*_m_ changes in the presynaptic excitatory neuron may modulate the size of excitatory postsynaptic potentials (EPSPs) in interneurons, leading to changes in their AP firing, which in turn alter the efficacy of their inhibition on neighboring neurons.

Cortical inhibitory interneurons show a huge diversity in their biochemical and physiological properties [18],[19]. Two distinct subtypes of interneurons, low-threshold spiking (LTS) neuron and fast spiking (FS) neuron, mediate the slow and the fast recurrent inhibition, respectively [27]–[29]. The LTS neuron receives EPSPs that show facilitation in response to a train of high-frequency stimuli of the presynaptic PC [30]–[32], generating APs with a long onset latency and evoking late-onset (slow) disynaptic IPSPs in its neighboring PCs [27],[29],[33]. The FS neuron (and some other inhibitory interneurons) receives EPSPs that show depression during high-frequency presynaptic stimulation [31],[34]. However, the high release probability of PC-FS synapses often allows discharges of the FS neuron in response to single APs in the PC, leading to time-locked early onset (fast) IPSPs in neighboring PCs [29],[35]. In this study, we sought to examine whether the slow and the fast recurrent inhibition meditated by these two distinct microcircuits (PC-LTS-PC and PC-FS-PC) were subjected to modulation in response to *V_m_* changes of the presynaptic PC.

We performed paired whole-cell recording (PC-PC, PC-LTS, and PC-FS) in rat somatosensory cortical slices and found that both the slow and the fast recurrent inhibition were indeed modulated by the presynaptic somatic *V*_m_ changes, and this modulation resulted from analog-mode signaling in excitatory synapses between PCs and interneurons. These results show an important role of analog communication in controlling the operation of cortical microcircuits and provide new insights into the cellular mechanisms underlying dynamic balance of cortical excitation and inhibition.

## Results

### *V*_m_-Dependent Modulation of Slow Recurrent Inhibition

**Figure 1:**
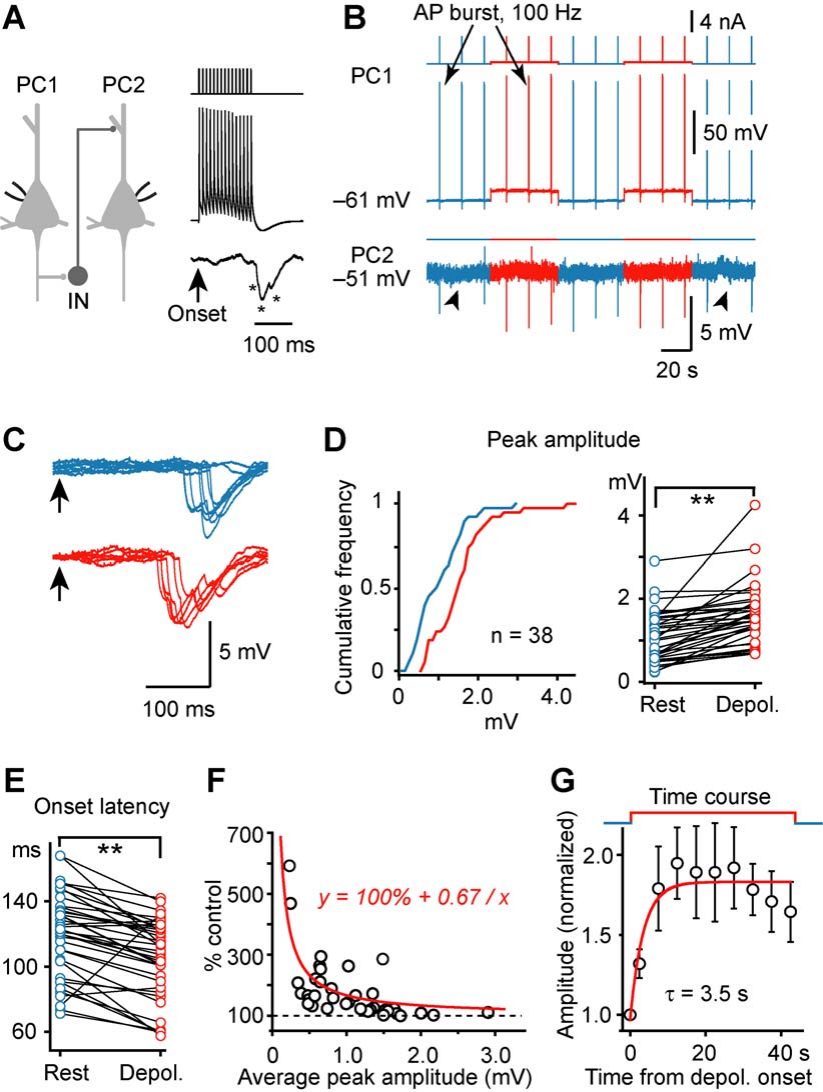
Modulation of slow (late-onset) disynaptic IPSP by presynaptic somatic *V*_m_. (A) Left, schematic diagram of the PC-PC paired recording (IN indicates those unidentified inhibitory interneurons that mediate the disynaptic IPSPs). Right, an AP burst (15 APs at 100 Hz) evoked by a train of current injection in PC1 induced a disynaptic response in PC2 with a long latency from the onset of the AP burst. * indicates individual IPSPs. (B) Example recording showing that presynaptic depolarization increased the amplitude of AP burst-induced disynaptic IPSPs. (C) Overlay of the IPSPs evoked at resting (blue) and depolarized (red) *V*_m_ in the presynaptic PC. Arrows indicate the onset of the AP train. Notice that presynaptic depolarization caused a reduction in failure, increased the amplitude, and shortened the latency of the disynaptic IPSPs. (D) Left, cumulative frequency distribution of the tested connections (*n* = 38 PC-PC pairs) by the average amplitude of disynaptic IPSP at resting (blue) and depolarized *V*_m_ (red); right, pooled results showing changes of the average amplitude at the two *V*_m_ levels in individual PC-PC pairs. (E) Pooled results (*n* = 38 pairs) showing that the onset latency of IPSPs was shortened by presynaptic depolarization. (F) The percentage increase was dependent on the average amplitude of disynaptic IPSPs (*n* = 38 pairs). Red line, hyperbolic fit. (G) Average time course of the facilitation in PC-PC pairs that showed significant increase in IPSP amplitude (*n* = 12 pairs tested). Error bars represent s.e.m. ** *p*<0.01.

We performed paired whole-cell recording from nearby layer-5 pyramidal cells (PCs, <100 µm apart) in acutely isolated rat somatosensory cortical slices. In response to stimulation of the PC with a burst of APs (70∼200 Hz), disynaptic IPSPs were observed in 19% of the pairs successfully tested (207/1,087 pairs), with 2% (23/1,087) exhibiting reciprocal IPSPs (see Methods, Figure 1A). Consistent with previous studies [27],[29], these IPSPs had a peak amplitude of 1.3±0.1 mV (s.e.m., *n* = 38 PC-PC pairs) and a long but rather precise onset latency (111±4 ms) following PC stimulation (15 APs at 100 Hz). The IPSPs were detected only when the presynaptic PC fired at a frequency higher than 50 Hz. Bath application of either CNQX (10 µM) or picrotoxin (50 µM) completely abolished these IPSPs (*n* = 8/8 PC pairs), consistent with the involvement of both excitatory and inhibitory transmission in these disynaptic responses. In our PC-PC paired recordings, a single AP or a burst of APs in one PC could evoke monosynaptic EPSPs but never triggered firing in postsynaptic PCs, suggesting no polysynaptic events involved in generating the disynaptic IPSPs.

To examine the *V*_m_-dependence of this slow (late-onset) recurrent inhibition, we manipulated *V*_m_ by injecting repetitive step-depolarizing currents (duration ∼45 s, at 90 s intervals) to induce subthreshold depolarization in the presynaptic PC. The magnitudes of disynaptic IPSPs were measured by applying trains of current pulses (duration: 1 ms) to evoke AP bursts (15 APs at 100 Hz) in the presynaptic PC at the resting and depolarized *V*_m_ (Figure 1B and 1C). In the majority of PC-PC pairs tested with this protocol (*n* = 38 pairs), we found that step presynaptic depolarization from a resting *V*_m_ (−64.6±0.6 mV) to a level near the firing threshold (−47.2±0.6 mV) significantly increased the average amplitude (*n* = 25/38 PC pairs tested, *p*<0.05) and integrated voltage area (mV×s, *n* = 28/38, *p*<0.05) of the disynaptic IPSPs and decreased the average onset latency and jitter (*n* = 23/38, *p*<0.05). Cumulative frequency distribution of the average amplitude (or total voltage area, unpublished data) showed a highly significant difference between the two *V*_m_ levels (*n* = 38 pairs, Figure 1D, *p*<0.01, Kolmogorov-Smirnov test). In these experiments, we noted that the extent of modulation of these IPSPs varied greatly from pair to pair. Comparison of the IPSP amplitude (or total voltage area) and onset latency at two different *V*_m_ levels of the same pairs also showed highly significant differences (Figure 1D and 1E, *p*<0.01, *t* test). Interestingly, the percentage increase in the peak amplitude (or total voltage area) of IPSPs found at the depolarized *V*_m_ levels decreased with increasing average peak amplitude. As shown in Figure 1F, the data could be well fitted by a hyperbolic function (y = 100%+0.67/x), which predicts a linear relationship between the IPSP amplitudes at depolarized *V*_m_ and those at resting *V*_m_ (y = x+0.53, with the slope fixed at 1, see Figure S1 and Discussion). In 12 PC-PC pairs, we varied the time difference between the depolarization onset and the first AP burst during the depolarization in order to examine the time course of the facilitation and found that the IPSP amplitudes progressively increase after depolarization with a time course τ of 3.5 s (single exponential fit, Figure 1G), consistent with the slow component of the EPSP facilitation induced by presynaptic depolarization found in monosynaptically connected PC pairs [26].

**Figure 2:**
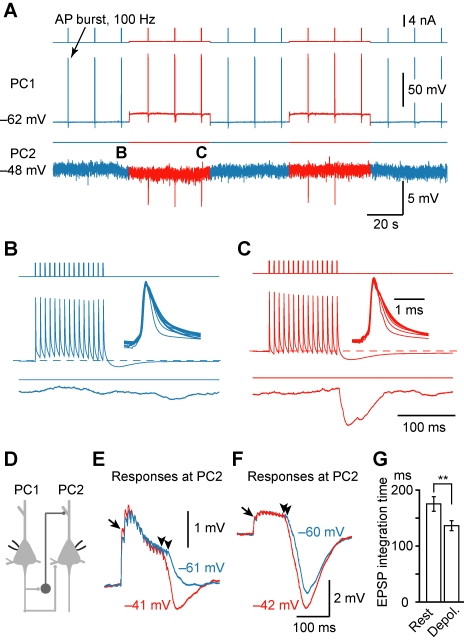
Presynaptic depolarization turns on recurrent inhibition and shortens the integration time of EPSPs. (A) Example PC-PC paired recording showing that the disynaptic IPSP only occurred at a depolarized *V*_m_. (B and C) Parts in (A) were expanded for clarity. Insets, overlay of the somatic APs during the train indicating that no AP failure occurred. (D) Schematic drawing of the recordings (for E–G) from PC-PC pair that had both the monosynaptic excitatory connections and the disynaptic inhibitory connections. (E) An example showing that disynaptic IPSP (average of 33 trials), which occurred only when the presynaptic *V*_m_ was depolarized, shortened the EPSP summation time (arrowheads). The arrow indicates a facilitated EPSP. (F) Similar example (average of 15 trials) as shown in (E) except that disynaptic IPSP occurred at both depolarized and resting presynaptic *V*_m_. Note the difference in the time window of EPSP summation (arrowheads). (G) Group data (*n* = 9 PC-PC pairs) indicated that presynaptic depolarization shortened the time window for EPSP integration. ** *p*<0.01.

Further analysis revealed that the disynaptic IPSP facilitation could be attributed in part to a decrease in its failure rate. In 33/35 PC pairs tested, IPSP failures occurred at both depolarized (by 18.0±0.8 mV) and resting presynaptic *V*_m_, but the average failure rate was lower under depolarized *V*_m_ (0.08±0.02) than that at resting *V*_m_ (0.25±0.03). Among all experiments (*n* = 123 PC pairs), nine showed complete failure of disynaptic IPSPs at resting *V*_m_ but detectable IPSPs at depolarized *V*_m_ (Figure 2A–C), suggesting that such modulation could not only change but also turn on recurrent inhibition. This abrupt appearance of disynaptic IPSPs and the shortened onset latency associated with presynaptic depolarization may narrow the time window of the integration of EPSPs. Indeed, in PC connections that had both monosynaptic EPSPs and disynaptic IPSPs, the EPSP summation time was shorter at depolarized *V*_m_ in comparison with that at resting *V*_m_ (136±9 ms versus 175±13 ms, *p*<0.01, *n* = 11, Figure 2D–G).

**Figure 3:**
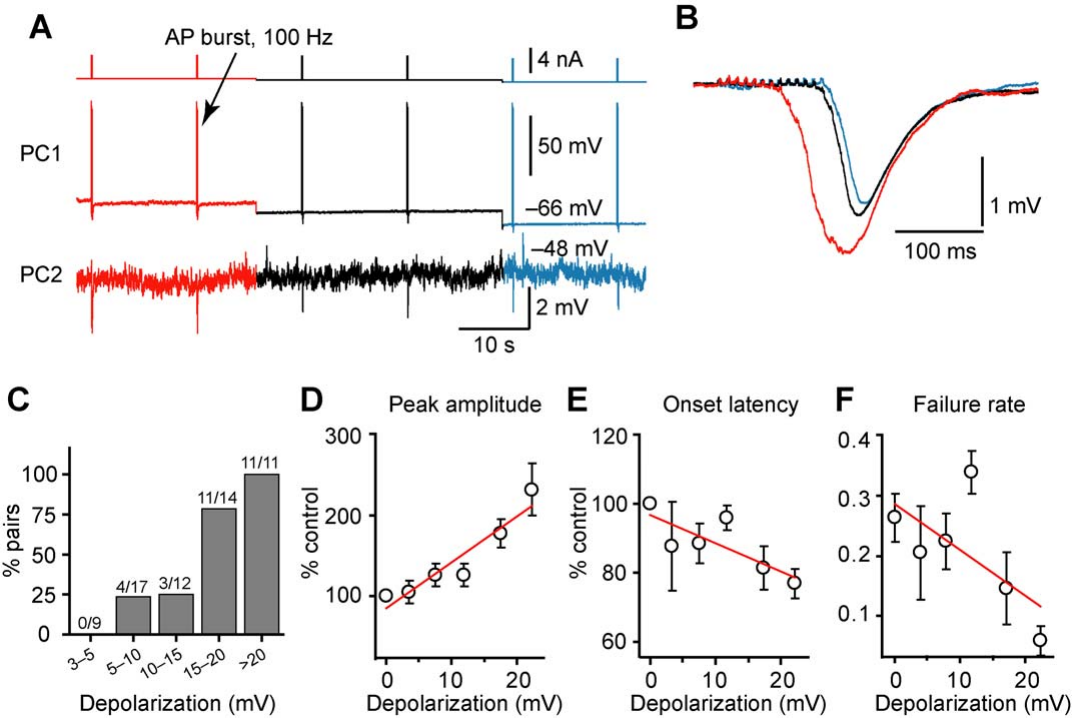
*V*_m_-dependence of disynaptic IPSP facilitation. (A) Example PC-PC paired recording showing that the amplitude of disynaptic IPSP was closely associated with presynaptic *V*_m_ levels. (B) Overlay of the averaged IPSPs at different presynaptic *V*_m_ levels. Notice the changes of the IPSP amplitude and onset latency. Same PC-PC pair as in (A). (C) Bar plot of percentage of pairs that showed facilitation of disynaptic IPSPs at different levels of presynaptic depolarization. Notice that depolarization progressively increased the percentage of pairs that exhibited facilitation. (D) Group data showing that the IPSP amplitude correlated closely with the level of depolarization. (E) Onset latency shortened with depolarizing *V*_m_. (F) Failure rate of the disynaptic IPSPs decreased with depolarizing *V*_m_. Red lines indicate linear fits of the data. Data were shown as mean ± s.e.m.

We next examined a range of presynaptic *V*_m_ in PC-PC pairs that showed IPSP facilitation to determine the threshold depolarization for inducing facilitation and the *V*_m_-dependence of facilitation (Figure 3A–B). None of the nine connections tested showed facilitation when the presynaptic PC was depolarized by only 3–5 mV. However, 5–10 mV depolarization resulted in IPSP facilitation in 24% (*n* = 4/17) of the connections tested. The percentage of pairs exhibiting facilitation increased to 100% for depolarization more than 20 mV (*n* = 11/11, Figure 3C). The IPSP amplitudes also increased and their onset latencies decreased with increasing depolarization of the PC, as compared to those observed at the resting *V*_m_. As shown in Figure 3D–E, for pairs that exhibited disynaptic facilitation (*n* = 20), the average IPSP amplitude (including failures) increased progressively with presynaptic depolarization (*r* = 0.95, 5.7% per mV), whereas the onset latency decreased accordingly (*r* = −0.80, 0.8% per mV). Again, the increase in IPSP amplitude was partially due to the decrease of failure rate (Figure 3F).

Together, these results show that a relatively small *V*_m_ shift of 5–10 mV of the presynaptic PC can alter the amplitude and the onset latency of disynaptic IPSPs received by its neighboring neurons, indicating a robust *V*_m_-dependent modulation of slow recurrent inhibition.

### Role of LTS Interneurons

We next investigated the mechanisms underlying this *V*_m_-dependent modulation. The late-onset disynaptic IPSP between excitatory PCs is known to be mediated by LTS interneurons [27],[29]. In PC-LTS paired recordings, a train of high-frequency APs in the PC results in facilitating EPSPs and AP generation in the LTS neuron, which in turn triggers IPSPs in the PC [27],[29]. We therefore examined whether the *V*_m_ changes in the presynaptic PC could modulate the magnitude of summated EPSPs and discharge probability of its postsynaptic LTS neuron.

**Figure 4:**
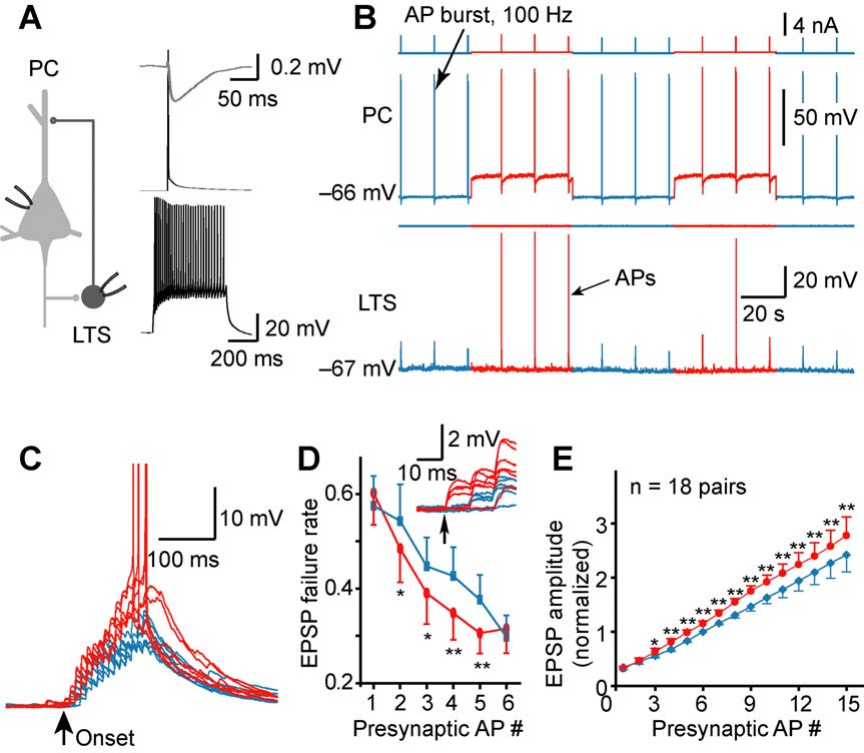
*V*_m_-dependent modulation of the slow recurrent inhibition is mediated by LTS interneurons. (A) Left, schematic diagram of the PC-LTS paired recording. Right, the characteristic firing pattern of the LTS was shown at the bottom (in response to a square pulse of 0.2 nA); single AP (middle) in the LTS could evoke an IPSP (top) in the PC. (B) Example recording from the PC-LTS pair shown in (A). Presynaptic depolarization (∼20 mV) increased the peak amplitude of the summated EPSPs (evoked by a burst of 15 APs at 100 Hz in the PC) and occasionally caused AP firing in the LTS. (C) Overlay of the summated EPSPs recorded at the LTS. Note the facilitated EPSPs. (D) The failure rate of the 2^nd^ to 5^th^ EPSPs decreased after presynaptic depolarization. Inset, example traces showing EPSP failures occurred at resting (blue) and depolarized *V*_m_ (red); arrow indicates the onset of the presynaptic AP train. (E) Group data (mean ± s.e.m., including EPSP failures) indicating the facilitation of individual EPSPs after presynaptic depolarization. Peak amplitudes of individual EPSPs were normalized to the 6^th^ EPSP at resting presynaptic *V*_m_. See also Figure S3.

In PC-LTS paired recordings (Figure 4A, see also Figure S2A–D), a burst of APs (15 APs at 100 Hz) initiated in the presynaptic PC caused significant synaptic facilitation that triggered spiking of the LTS cell (Figure 4B–C). Consistent with previous studies [27],[29], the spiking probability and the onset of LTS spiking depended on the number and the frequency of presynaptic APs (unpublished data). To reveal the effect of presynaptic *V*_m_ on the summated EPSPs, we hyperpolarized the LTS cell to prevent its spiking. Steady depolarization of the presynaptic PC from the resting *V*_m_ to a level near the firing threshold significantly increased the peak amplitude of the summated EPSPs (from 4.4±0.7 to 5.2±0.9 mV, *n* = 18, *p*<0.01) and the total integrated area associated with the EPSPs (from 0.6±0.1 to 0.8±0.2 mV×s, *p*<0.01). Close examination of the individual EPSPs revealed that the failure rate of the 2^nd^ to 5^th^ EPSPs was significantly lower when presynaptic PC was depolarized, as compared to that found at the resting *V*_m_ (*p*<0.05, Figure 4D). Peak amplitudes (measured from the baseline before the train) of individual EPSPs during the train were also significantly increased (Figure 4E, Figure S3), as reflected by the increased slope in the plot of normalized EPSP amplitude versus the AP number (from 0.12 to 0.15 per AP, *n* = 18 PC-LTS pairs).

**Figure 5:**
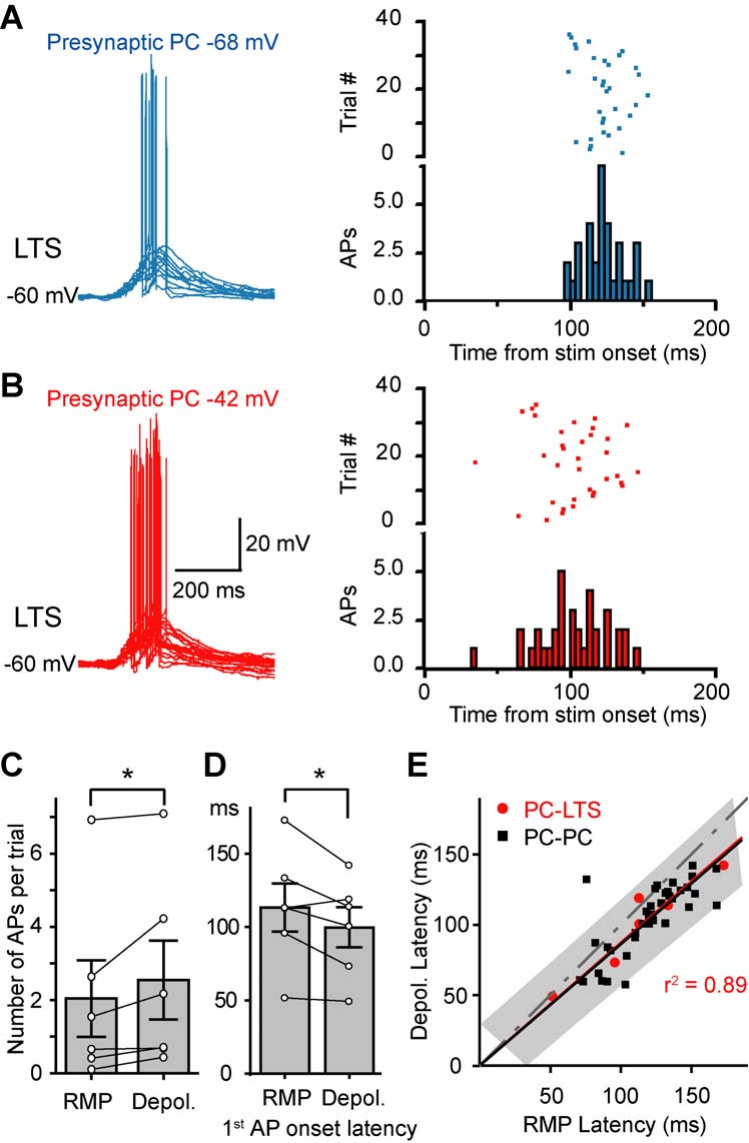
PC depolarization increases the number and reduces the onset latency of LTS APs. (A) Left, overlay of example postsynaptic responses of LTS to a train of presynaptic APs at resting presynaptic *V*_m_. Right, rasters and peristimulus histogram showing the number and timing of APs in LTS across trials. (B) Same cell as in (A). Presynaptic *V*_m_ was depolarized to −42 mV. Notice the increase in number of APs and the decrease in AP onset latency. (C) Presynaptic depolarization significantly increased the number of APs per trial in LTS cells. (D) Comparison of the 1^st^ AP onset latency at resting versus depolarizing *V*_m_. mean ± s.e.m. * *p*<0.05. See also Figure S4. (E) Plot of the onset latency of disynaptic IPSPs in PC-PC pairs (black symbols; data from Figure 1) and LTS spiking in PC-LTS pairs (red symbols; data from panel D) at depolarized *V*_m_ as a function of those at resting *V*_m_. Note that the majority of the points lie below the dotted line (slope = 1), indicating the latencies at depolarized *V*_m_ were shorter than those at resting *V*_m_. Gray area indicates the 95% prediction bounds for IPSP latencies. Note that the points for LTS spiking latencies fall in this prediction bounds. The black and red lines are the linear regression fits for the IPSP and LTS spiking latencies, respectively.

Next, we compared the spiking probability of the postsynaptic LTS cell before and after the presynaptic *V*_m_ shift from resting to depolarized levels. Summated EPSPs evoked by trains of presynaptic APs (15 APs at 100 Hz) triggered AP generation in some of the LTS neurons recorded at resting *V*_m_ (*n* = 6/22 PC-LTS pairs; Figure 4B–C and Figure 5). In these six pairs that exhibited LTS spiking, the shift of *V*_m_ in the presynaptic PC from resting to a level near the firing threshold resulted in an increase in numbers of APs per trial in LTS cells (from 2.0±1.0 to 2.5±1.1, *p*<0.05; Figure 5A–C) and a decrease in the onset latency (from 113.3±16.4 to 99.7±13.7 ms, *p*<0.05; Figure 5A–B and D) and jitter of spiking (from 25.2±3.7 to 21.2±2.9 ms, *p* = 0.055). Similar results could be obtained even when presynaptic PC was stimulated at low intensities (10 APs at 20 Hz, Figure S4). Plot of the onset latency of LTS spiking and disynaptic IPSPs (shown in Figure 1E) at depolarized *V*_m_ as a function of that at resting *V*_m_ revealed a close correlation between them (Figure 5E), indicating that the decrease in IPSP latency resulted from the early spiking of LTS cells. Taken together, these results correlate well with the findings on the *V*_m_-dependent modulation of slow disynaptic IPSPs described above (see Figure 1) and indicate that PC depolarization may recruit more LTS cells and/or trigger more firing in these cells, thus causing more inhibition in their neighboring PCs.

In these experiments, we also found that single APs in LTS cells evoked IPSPs in PCs with a failure rate of 0.19±0.04 and an average amplitude of −0.43±0.14 mV (*n* = 19 LTS-PC pairs) at a holding potential of approximately −50 mV. Consistent with their distal input location at the apical dendrite, these IPSPs had a reversal potential of −80.2±1.9 mV (*n* = 6 pairs). The rise time and the decay time constant (τ) were 7.25±0.82 and 58.6±8.3 ms, respectively (*n* = 16 pairs). These basic kinetics were different from those of FS-PC IPSPs (rise, 4.76±0.64 ms; decay, 85.2±15.5 ms; *n* = 28 pairs), which had a reversal potential of −70.8±1.4 mV (*n* = 4 pairs). In 13/52 LTS-PC pairs tested, we observed reciprocal connections (Figure 4A), suggesting that the *V*_m_-dependent modulation of disynaptic IPSPs could directly influence the feedback inhibition of the presynaptic PC, in addition to the inhibition of other downstream PCs.

### *V*_m_-Dependent Modulation of Fast Recurrent Inhibition

**Figure 6:**
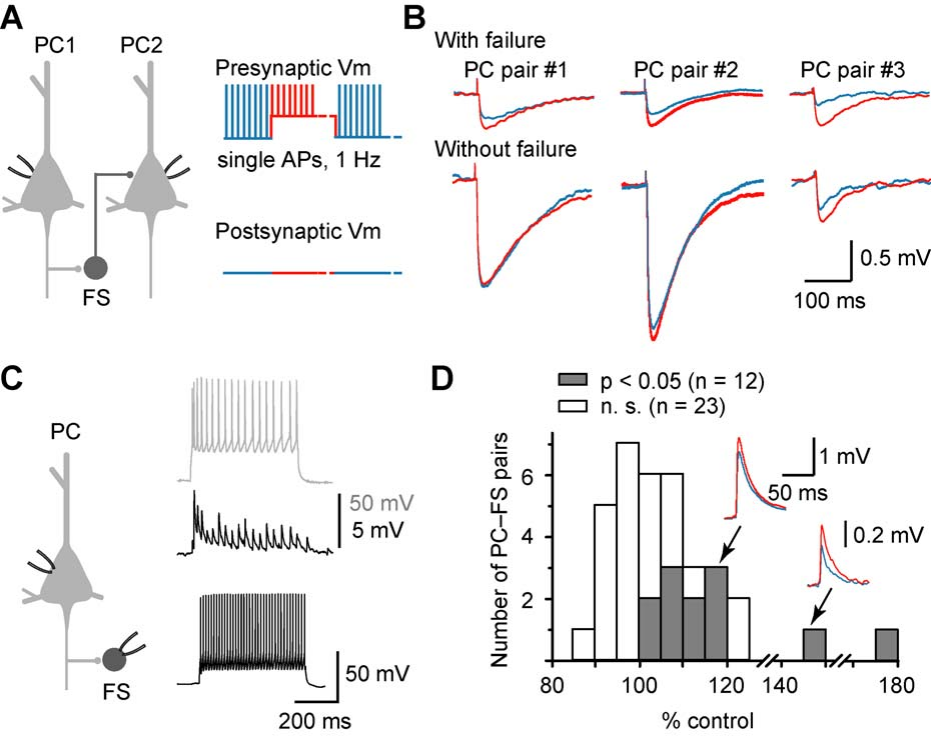
*V*_m_-dependent modulation of fast (early-onset) disynaptic IPSP. (A) Schematic diagram showing the PC-PC paired recording and the protocol of stimulation. Brief injection (1 ms) of depolarizing current pulses to the presynaptic PC evoked single APs at a rate of 1 Hz, while periodic constant current injection caused ∼20 mV depolarization from the resting *V*_m_. The resting and depolarized periods were ∼15 s each. The dotted lines indicate a break in the time axis. (B) PC-PC paired recordings tested with the protocol shown in (A). Traces (average of at least 300 trials) from three pairs showing that the average disynaptic IPSP at depolarized *V*_m_ (red) were larger than that at resting *V*_m_ (blue) when IPSP failures were included. (C) PC-FS paired recording. Left: recording configuration. Right: bottom trace showing the non-adapting fast-spiking pattern of the recorded FS neuron in response to a current pulse (0.4 nA); the middle trace indicates the depressing EPSPs in FS neuron in response to a train of APs in the PC (0.3 nA, top trace). (D) Group data from PC-FS pairs using similar protocol as in (A). Significant EPSP facilitation was observed in 12 out of 35 pairs. Inset: example traces (average of at least 300 trials) from two PC-FS pairs.

To investigate whether the fast disynaptic inhibition mediated by interneurons that receive depressing excitatory inputs is also *V*_m_-dependent, we analyzed the PC-PC pairs that exhibited fast (early onset) IPSPs in response to a single presynaptic AP. Consistent with previous reports [27]–[29], we found that the probability of success in detecting fast disynaptic IPSPs was very low. Only 7/1,103 PC-PC pairs tested bi-directionally exhibited fast disynaptic IPSPs, and three out of these seven pairs showed *V*_m_-dependent modulation. Presynaptic depolarization of ∼18 mV from the resting *V*_m_ (−63 mV) substantially reduced the failure rate of evoking IPSPs in these three pairs (from 0.78 to 0.67, 0.85 to 0.78, and 0.63 to 0.36, respectively; Figure 6A–B). This reduced failure rate is consistent with our hypothesis that the depolarized *V*_m_ in the PC elevated the EPSP amplitude and increased the spiking probability of the FS cell. When the average amplitude of IPSPs (failure excluded) evoked by single APs was measured, we found that it was unchanged in the first two pairs (from 1.42±0.05 to 1.44±0.04 mV, *p* = 0.4; from 2.0±0.1 to 2.1±0.1 mV, *p* = 0.2). This supports the notion that disynaptic modulation was mainly due to changes in the firing probability of the interneurons. However, we found surprisingly that the average IPSP amplitude observed in the third pair was significantly increased from 0.62±0.02 to 0.75±0.02 mV (*p*<0.01) even with failure excluded. Given the low probability of observing fast disynaptic IPSPs (*n* = 7/1,103 PC-PC pairs), the recruitment of two interneurons in this case is unlikely. However, it is possible that triggering of two APs (instead of one) in the interneuron during presynaptic depolarization could account for the remaining increase in IPSP amplitude after failure exclusion. The precise mechanism for these observations remains to be further examined.

The interneurons that mediate these fast disynaptic IPSPs are most likely FS neurons, which receive depressing EPSPs in response to high-frequency presynaptic stimulation [31],[34]. We therefore recorded PC-FS pairs to examine whether EPSPs are indeed subjected to modulation by the *V*_m_ levels of PCs. Consistent with previous findings [25],[26], presynaptic depolarization of 15–20 mV significantly increased the average amplitude of the single AP-triggered EPSPs (single AP at 1 Hz) in about one-third of the pairs tested (*n* = 12/35 PC-FS pairs, Figure 6C–D). These elevated EPSPs may increase the spiking probability of FS cells, thus reducing the failure rate in evoking IPSPs in their postsynaptic PCs. Together, these results show that fast recurrent inhibition is also *V*_m_-dependent, resulting from the *V*_m_-dependent analog signaling in excitatory synapses between PCs and interneurons.

### *V*_m_-Dependent Modulation of Monosynaptic IPSP

**Figure 7:**
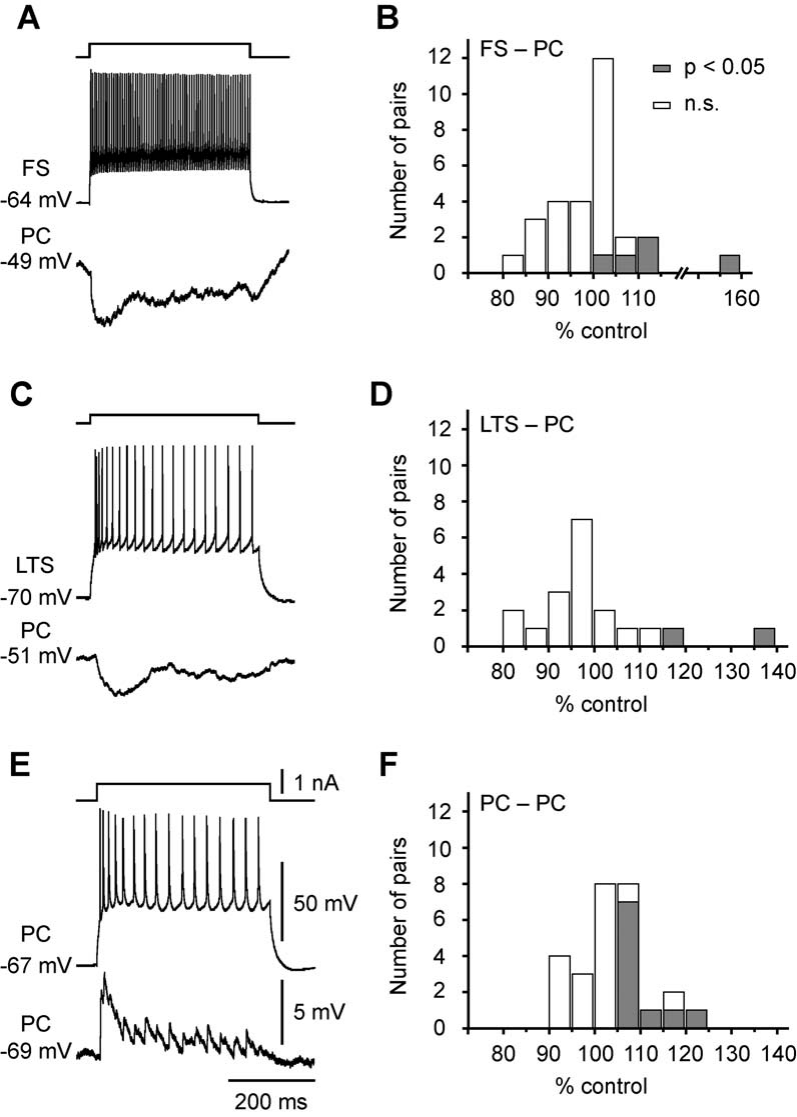
*V*_m_-dependent modulation of monosynaptic IPSPs and PC-PC EPSPs. (A) Example recording showing inhibitory connection in a FS-PC pair. (B) Group data from FS-PC pairs. Filled boxes, pairs that showed significant increase in the average peak amplitude of IPSPs evoked by single APs (1 Hz, similar protocol as in Figure 6A) after presynaptic depolarization (∼20 mV). Open boxes, pairs without significant facilitation. (C) Example recording showing inhibitory connection in a LTS-PC pair. (D) Group data for LTS-PC pairs. (E) Example recording showing excitatory connection in a PC-PC pair. (F) Group data for PC-PC pairs.

Since cortical states affect both PCs and inhibitory interneurons [8],[11], we next examined whether this *V*_m_-dependent analog signaling occurs at inhibitory synapses. In the FS-PC and LTS-PC pairs that showed monosynaptic inhibitory connections, we depolarized the presynaptic FS or LTS cells from resting *V*_m_ (∼−70 mV) to a level near the firing threshold and found that monosynaptic IPSPs evoked by single APs (using similar protocol as that shown in Figure 6A, also see [26]) showed no significant change in basic kinetics of IPSPs (FS-PC: rise time from 4.76±0.64 to 4.16±0.50 ms and decay τ from 85.2±15.5 to 62.0±3.6 ms, *n* = 28 pairs; LTS-PC: rise time from 7.25±0.82 to 7.04±0.72 ms and decay τ from 58.6±8.3 to 60.9±7.4 ms, *n* = 16 pairs; *p*>0.05); however, the average amplitude of IPSPs was significantly enhanced in a small subpopulation of tested pairs. The percentages of FS-PC and LTS-PC pairs that showed IPSP facilitation in response to presynaptic depolarization were 17.2% (*n* = 5/29) and 10.5% (2/19), respectively, which were smaller than that for PC-PC pairs (37.0%, *n* = 10/27) exhibiting EPSP facilitation (Figure 7). The lower probability of finding *V*_m_ modulation in LTS-PC pairs than in FS-PC pairs may result from the differences in the location of the inhibitory synapses. The LTS cells send their axons to superficial layers and form synapses onto the distal apical dendrites of PCs, while FS cells mainly target the perisomatic region of PCs. Thus, *V*_m_ changes at the soma may decay more substantially when arriving at axon terminals in LTS cells than FS cells to influence synaptic transmission. Taken together, these results demonstrate that monosynaptic IPSPs are also subjected to modulation by *V*_m_ changes in presynaptic interneurons in a small subpopulation of inhibitory connections.

### Role of Axonal K_v_1 Channels

The above experiments showed that both fast and slow recurrent inhibition were subjected to modulation by *V*_m_ changes in PCs, resulting from the *V*_m_-dependent analog-mode signaling in PC-interneuron excitatory synapses. A rapidly activating but slowly inactivating axonal K^+^ current, known as D-current [36], has been shown to regulate the axonal AP duration and potentially contribute to the *V*_m_-dependent modulation of the EPSP amplitude [37],[38]. We thus further investigated the role of axonal D-currents in *V*_m_-dependent modulation of recurrent inhibition in cortical microcircuits.

**Figure 8:**
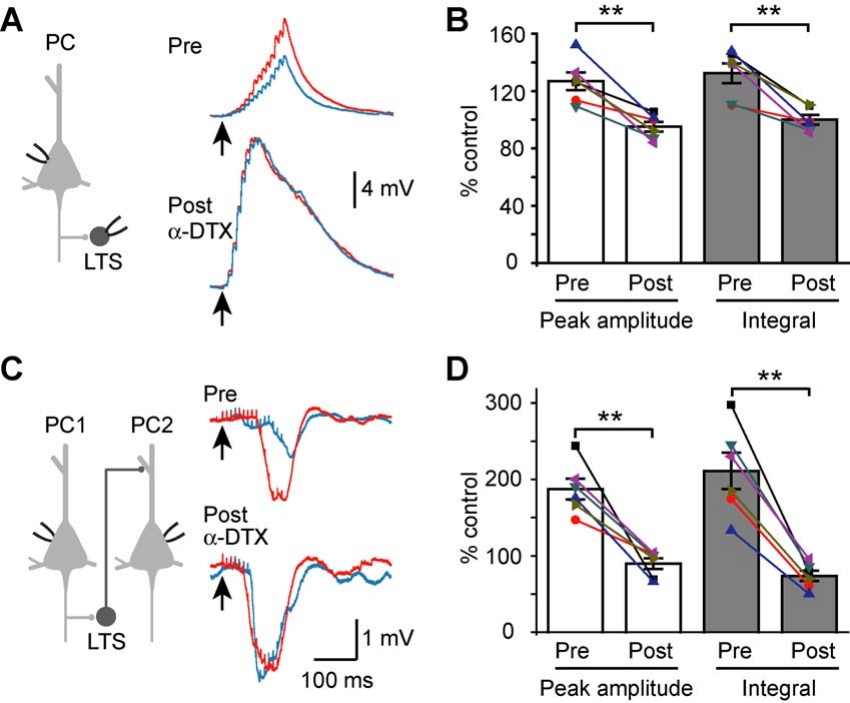
Role of K_v_1 channels in the *V*_m_-dependent modulation of disynaptic IPSPs. (A) Left, schematic diagram of the PC-LTS pair recording. Right, an example recording showing that bath application of α-DTX (100 nM) increased the size of disynaptic IPSPs and occluded the facilitation induced by PC depolarization. Blue and red traces are the averaged EPSPs evoked by trains of APs (same protocol as in Figure 4) at resting and depolarized *V*_m_, respectively. Note the depolarization-induced facilitation of the summated EPSPs before α-DTX application. (B) Group data (*n* = 6) showing that α-DTX diminished the depolarization-induced increases in the peak amplitude and the integral of the summated EPSPs. (C) Similar recording as shown in Figure 1. Note that α-DTX not only mimicked the depolarization-induced facilitation but also occluded the effect of *V*_m_ changes. (D) Group data (*n* = 6) showing that α-DTX blocked the *V*_m_ shift-induced changes in the peak amplitude and the integral of disynaptic IPSPs. ** *p*<0.01. See also Figure S5.

Consistent with previous findings, bath application of α-dendrotoxin (α-DTX, 100 nM) blocked the PC depolarization-induced facilitation of the summated EPSPs in LTS cells. In six PC-LTS pairs, depolarization of ∼20 mV in the PC caused a significant increase in the peak amplitude (126.9%±6.2% of the control, *p*<0.05) and the integrated area (132.3%±7.0%, *p*<0.05) of the summated EPSPs evoked by 15 APs at 100 Hz, and this increase was blocked in the presence of α-DTX (peak amplitude, 95.0%±3.5%; voltage area, 100.0%±3.4%; Figure 8A–B). In PC-PC pairs, we found that α-DTX application significantly increased the amplitude and integrated area of the disynaptic IPSP to 187%±14% (*p*<0.01) and 211%±24% (*p*<0.01), respectively, and shortened the onset latency to 86.1%±8.1% (*p*<0.05, *n* = 6). These effects suggest that D-current inhibition is sufficient to facilitate disynaptic IPSPs. Furthermore, after α-DTX application, 20 mV depolarization of the presynaptic PC had no significant additional facilitation in the amplitude (89.7%±7.1%, *p* = 0.31), total integrated area (73.7%±6.9%, *p* = 0.9), and onset latency (99.2%±3.4%, *p* = 0.46, Figure 8C–D) of disynaptic IPSPs, indicating that the effect of α-DTX had occluded that of *V*_m_ changes. Similar results were obtained with the application of a low concentration of 4-AP (50 µM, Figure S5). These results support the hypothesis that inhibition of axonal D-current in the presynaptic PC mediates the *V*_m_-dependent modulation of recurrent inhibition.

## Discussion

In this study, we show that the magnitude of recurrent inhibition of PCs depends on their *V*_m_ levels; this *V*_m_-dependent modulation occurs in both LTS and FS cell-mediated disynaptic recurrent connections. We provide the first evidence showing that presynaptic *V*_m_-dependent analog communication occurs at excitatory synapses between PCs and interneurons and mediates the *V*_m_-dependent modulation of recurrent inhibition. For slow disynaptic IPSPs, depolarization in presynaptic PCs increases the amplitude of AP burst-induced summated responses in postsynaptic LTS neurons, thus increasing the number and decreasing the latency of their AP discharges, which in turn enhance the amplitude and reduce the onset latency and jitter of slow IPSPs in neighboring PCs. Similarly, modest depolarization in PCs enhanced single AP-induced fast disynaptic IPSPs in neighboring PCs presumably due to a *V*_m_-dependent increase in the size of EPSPs in FS neurons. Together, these results reveal that PC *V*_m_-dependent modulation of cortical inhibition is a key strategy through which the cortex efficiently and dynamically maintains the excitation-inhibition balance, a condition critical for cortical information processing.

### Mechanisms for *V*_m_-Dependent Modulation of Inhibition

Recent studies have discovered that the PC-LTS-PC microcircuit mediates the slow (late-onset) recurrent inhibition in somatosensory and other cortices [27],[29],[39]. The somatostatin-positive LTS interneuron is the key player in this microcircuit. It receives EPSPs that show facilitation in response to a train of presynaptic stimuli that may initiate firing of APs, which in turn evoke IPSPs in its postsynaptic PCs [27],[29],[33]. Generation of these disynaptic IPSPs depends on the number and frequency of APs in PCs. Our results show that the presynaptic *V*_m_ is also a powerful determinant for controlling the strength and timing of disynaptic IPSPs—a few mV depolarization (>5 mV) can cause substantial IPSP facilitation (Figure 3). More importantly, stronger depolarization could not only modulate the amplitude of existing disynaptic IPSPs but also turn on silent recurrent connections (Figure 2). Further analysis showed a close relationship between the magnitude of IPSP facilitation and the extent of presynaptic depolarization in PCs, consistent with the requirement of excitation-inhibition balance during elevated network activity [1],[3]. The abrupt occurrence and the facilitation of disynaptic IPSPs may result from the increases in the spiking probability or the number of APs in LTS interneurons (Figures 4 and 5). Since the inhibitory synaptic strength at synapses between the newly recruited LTS interneurons and the postsynaptic PC should not depend on the amplitude of existing IPSPs, we expect that the net increase does not depend on the baseline IPSP amplitude. This was supported by the finding that the data shown in Figure 1F were well fitted by a hyperbolic function, which predicts a linear function between IPSP amplitudes at depolarized *V*_m_ and those at resting *V*_m_ (see Figure S1).

The PC-FS-PC microcircuit is a potential candidate mediating the fast (early-onset) recurrent inhibition [27],[29]. In contrast to LTS cells, FS neurons receive EPSPs that show depression in response to presynaptic high-frequency APs [31],[34]. A single presynaptic AP can trigger the FS neuron to discharge once and subsequently evoke a unitary IPSP at neighboring PCs [28],[29]. Consistent with the findings reported previously [27],[29], we observed fast disynaptic IPSPs in PC pairs at a very low frequency. In the PC pairs that showed depolarization-induced facilitation, we found that the facilitation resulted mainly from a reduced IPSP failure rate (Figure 6B), consistent with an increased EPSP amplitude and spiking probability of FS neurons. Thus, the same mechanism underlies *V*_m_-dependent modulation of disynaptic IPSPs in both types of microcircuits.

Consistent with previous studies [25],[26], our results showed that *V*_m_ changes in presynaptic PC modulated the sizes of EPSPs in both the LTS and the FS interneurons (Figure 4 and Figure 6). The underlying mechanism may depend on unique intrinsic properties of axonal ion channel subtypes. Recently, a low-threshold, slowly inactivating K^+^ current (known as D-current [36], mediated by K_v_1 alpha subunits) was recorded at the PC axons and was shown to selectively control the duration of axonal APs (instead of somatic APs) and the depolarization-induced facilitation of EPSPs [37],[38]. Consistently, our results demonstrated that the inhibition of K_v_1 channels was by itself sufficient to increase the size of summated EPSPs in PC-LTS and of disynaptic IPSPs in PC-PC pairs. Furthermore, it also occluded the effects of *V*_m_ at PC-LTS synapses and disynaptic transmission between PCs. Depolarization in the presynaptic cell could prolong axonal APs as well as activate presynaptic Ca^2+^ channels, thereby enhancing synaptic transmission by increasing the presynaptic background Ca^2+^ concentration and/or AP-triggered Ca^2+^ influx [40]–[42]. Indeed, high concentrations of EGTA could drastically reduce the success rate of EPSP facilitation induced by presynaptic somatic-depolarization [26] (but see [25]), suggesting that background Ca^2+^ may also contribute to the modulation of disynaptic IPSP. Inhibitory interneurons contain many kinds of Ca^2+^-binding proteins, such as parvalbumin, calretinin, and calbindin; they could function as Ca^2+^ buffer and thus may prevent the *V*_m_-dependent modulation of inhibitory transmission. Indeed, our results showed that the percentages of inhibitory connections (including FS-PC and LTS-PC pairs, Figure 7) showing *V*_m_ modulation are far less than those of excitatory connections (including PC-PC and PC-interneuron pairs, Figures 6 and 7). Whether the intracellular Ca^2+^-binding proteins may be responsible for regulating analog-mode signaling at inhibitory synapses remains to be further examined.

Subthreshold *V*_m_ changes in the soma spread down the axon with a length constant of 400–800 µm [25],[26],[37],[43]. Over 150 putative synaptic boutons are distributed at axon collaterals within 500 µm of the cell body of layer-5 PCs [26]. Boutons in remote axon terminals may not be affected by somatic *V*_m_ changes. This may explain why not all recurrent inhibitory connections were subjected to the *V*_m_-dependent modulation and why the percentages of LTS-PC pairs showing *V*_m_ modulation are smaller than those of FS-PC pairs. In comparison with FS cells that target the perisomatic region of their neighboring PCs, LTS cells in layer 5 send their axons to superficial layers and innervate distal apical dendrites of nearby PCs. Therefore, *V*_m_ modulation should be weaker in LTS-PC than in FS-PC synapses. Whether such modulation is indeed spatially confined to local circuits within the range of axonal spread of somatic depolarization, or alternatively only specific cortical microcircuits are modulated, remains to be further determined.

### Physiological Significance

The *V*_m_-dependent modulation of recurrent inhibition described here may serve several distinct functions. First, it may contribute to maintaining a dynamic excitation-inhibition balance at different cortical activity states appropriate for diverse behavioral conditions [8],[10],[11],[44]. For example, when *V*_m_ depolarizes during an active but relatively stable cortical state, e.g., the “Up” state, the inhibitory conductances due to recurrent connections increase to match the elevated excitatory conductances [1],[3],[4]. A recent work revealed that excitation-inhibition balance is also instantaneously controlled with a millisecond precision during spontaneous and sensory-evoked activities [2] and disruption of this balance causes dysfunction of the network [3],[9], leading to various disorders such as epileptic seizures [45],[46] and schizophrenia [47]. Second, the *V*_m_-dependent modulation of recurrent inhibition may also contribute to rapid transitions between “Up” and “Down” states [1],[3] that are important for gain modulation of synaptic and sensory inputs [1],[12],[13],[16],[17],[48],[49]. For example, transient excitation-inhibition imbalance caused by abrupt changes in *V*_m_-dependent recurrent inhibition, e.g., unsilencing of recurrent connections (Figure 2A–C) induced by *V*_m_ fluctuation at the depolarized *V*_m_ levels, could cause a switch from “Up” to “Down” state. Third, the shortening of IPSP latency associated with *V*_m_-dependent facilitation of recurrent inhibition (Figure 2D–G) may regulate the time window of integration of excitatory inputs, therefore providing a mechanism for *V*_m_-dependent feedback control of the timing of spike initiation in PCs [50]–[52].

To conclude, we have shown that both slow and fast recurrent inhibition is susceptible to modulation by the *V*_m_ changes of PCs. These results demonstrate a circuit function of *V*_m_-dependent modulation of excitatory transmission (analog-mode signaling). Whether such *V*_m_-dependent modulation is universal among all cortical circuits and whether it plays an important function in regulating circuit dynamics in behaviorally relevant conditions remain to be examined.

## Materials and Methods

### Ethics Statement

The use and care of animals complied with the guidelines of the Animal Advisory Committee at the Shanghai Institutes for Biological Sciences.

### Slice Preparation

We anesthetized the animal (15∼18-d-old Sprague-Dawley rats) with sodium pentobarbital (30 mg kg^−1^) before decapitation. The brain was quickly dissected out and immersed in an ice-cold oxygenated (95% O_2_ and 5% CO_2_) slicing solution in which the NaCl was substituted with sucrose (213 mM) and dextrose was reduced to 10 mM. We cut parasagittal slices (350 µm) of somatosensory cortex in this solution with a Leica microtome (VT-1000S) and immediately transferred to an incubation beaker filled with aerated normal artificial cerebrospinal fluid (ACSF) containing (in mM): NaCl 126, KCl 2.5, MgSO_4_ 2, CaCl_2_ 2, NaHCO_3_ 26, NaH_2_PO_4_ 1.25, and dextrose 25 (315∼325 mOsm, pH 7.3). Slices were incubated at 34.5°C for at least 45 min, then at room temperature until use. Visualization of cortical layers and neurons were made with an upright infrared-DIC microscope (BX51WI, Olympus) equipped with an infrared camera (OLY-150). All experiments were done at a temperature of 35.5–37°C.

### Electrophysiological Recordings

We obtained dual whole-cell recordings from layer-5 PCs and inhibitory interneurons (LTS and FS neurons) using Multiclamp 700B amplifiers (Molecular Devices). Patch pipettes were prepared with a P-97 microelectrode puller (Sutter Instruments) and filled with an internal solution containing (in mM) KGluconate 140, KCl 3, MgCl_2_ 2, Na_2_ATP 2, BAPTA 0.025, and HEPES 10 (pH 7.2 with KOH, 280∼290 mOsm). Patch electrodes had an impedance of 3–6 MΩ. For tracing and labeling the recorded neurons, we added Alexa Fluo 488 (100 µM) and biocytin (0.2%) to the pipette solution. We identified the recorded layer-5 neurons through their unique somatic and dendritic morphology under the DIC and fluorescent microscope and their distinct firing patterns. The total time the cell was exposed to fluorescence was kept to less than 10 s to minimize cell damage. The PCs could be easily distinguished from interneurons because of their thick apical dendrite and large pyramid-shaped somata. The FS neurons were identified through their non-adapting and fast-spiking (300–500 Hz in response to current stimulation, Figure 6 and Figure S2) firing properties and their “noisy” resting *V*_m_ constantly bombarded with large-amplitude EPSPs. The LTS neurons (Figure 4) were classified through their low-threshold regular firing patterns with initial accelerating then decelerating discharges in response to step current injection (see also Figure S2). After electrophysiological recording, the neurons were further identified using DAB-staining. The intrinsic properties of individual neurons and the properties of synaptic connections between PCs and FS and LTS neurons (<100 µm apart) were examined as soon as a dual or triple recording was achieved. We injected negative (−0.1∼−0.5 nA) and positive current pulses (0.1∼1.5 nA, 500 ms) to examine the input resistance and firing patterns of each neuron. To test for synaptic connections, we injected 1-ms current pulses (10∼20 pulses) at a frequency of 20∼200 Hz to each PC to evoke a train of APs every 15 s while monitoring the *V*_m_ changes in other PCs or interneurons. Unless otherwise stated, for data analysis and figures, we normally evoked a train of 15 APs at 100 Hz through current injection in the presynaptic PC. In most experiments, Cl^−^ concentration in the recording pipette was 7 mM, and the calculated reversal potential for Cl^−^ was −74 mV. Disynaptic IPSPs recorded between PCs with this pipette solution were hyperpolarizing potentials at a depolarized postsynaptic *V*_m_ (normally depolarized from resting to ∼−46 mV with DC current injection). We injected constant DC current to evoke intermittent depolarizing and hyperpolarizing *V*_m_ levels (∼10–20 mV, ∼45 s each) in the presynaptic PC while keeping the postsynaptic *V*_m_ constant. In the recordings examining the monosynaptic connections between PC and FS neurons, we used a high concentration of Cl^−^ (75 mM) in the pipette solution. To test for the presynaptic *V*_m_-dependent modulation of monosynaptic EPSPs in PC-FS pairs and fast disynaptic IPSPs in PC pairs, we only injected brief pulses to presynaptic PC to evoke single APs at 1 Hz on top of the intermittent depolarizations and hyperpolarizations. In most of our recordings, synaptic responses were stable and could be recorded up to 1–2 h after obtaining whole-cell recordings without any apparent rundown. Data were discarded if the evoked IPSPs and EPSPs showed significant rundown, as shown by a statistically significant change in the amplitude of the IPSP or EPSP between the first and last third of the interspersed control periods (when the presynaptic PC was at resting *V*_m_). The *V*_m_ values were not corrected for the liquid junction potential (15 mV).

During the whole period of recording, access resistance was monitored frequently; recordings with access resistance higher than 25 MΩ were discarded. Bridge balance and capacitance neutralization were carefully adjusted before and after every experimental protocol. We collected the electrophysiological data using a Micro 1401 digitizer and Spike 2 software (Cambridge Electronic Design, Cambridge, UK). After a recording was completed, the slice was transferred to 4% paraformaldehyde in 0.1 M phosphate buffer for subsequent immunostaining and visualization.

CNQX (AMPA receptor antagonist), picrotoxin (PTX, GABA_A_ receptor antagonist), α–dendrotoxin (α–DTX, K_v_1 channel blocker), and 4-aminopyridine (4-AP, D-current blocker when applied at low concentrations) were applied through bath perfusion. Their concentrations were indicated in the text.

### Data Analysis

We performed all computations using Spike 2 and MATLAB (MathWorks, Bethesda, MD). The significance of differences between the cumulative frequency distributions was determined by Kolmogorov-Smirnov test using original disynaptic IPSPs. We used Student's *t* test to test the significance of differences in peak amplitude, integrated voltage area, and onset latency between resting and depolarized presynaptic *V*_m_ in individual pairs. Values were presented as mean ± standard error in the figures as well as in the main text.

For disynaptic IPSPs, the peak amplitude was the difference between the peak value and the average baseline *V*_m_ (2 s prior to the stimuli onset), whereas the integral voltage area was the curve area underlying the responses. The onset latency was the time difference between the response onset and the beginning of presynaptic stimulation. For comparison of these values at different *V*_m_, we normalized the values to those obtained at the baseline *V*_m_ for each pair and then performed the statistical tests. To identify the EPSP failures in PC-LTS pairs, we first averaged the EPSPs evoked by presynaptic AP trains and selected an EPSP template from the average trace, then performed a correlation test between the voltage trace after each AP and the template EPSP. A failure was identified if the correlation coefficient was lower than 0.8. Considering that the first five EPSPs during the train had a high failure rate and most of them showed significant differences in the failure rates at different *V*_m_ (Figure 4D), we therefore chose the 6^th^ EPSP amplitude (measured from the baseline before the train, see Figure S3) as a reference for normalization. The peak amplitude of each EPSP during the train was normalized to the 6^th^ EPSP for each PC-LTS pair and then averaged for group data presentation (Figure 4, Figure S3). For the monosynaptic connections from PC to FS neurons (or from interneurons to PCs), we calculated the average EPSP (or IPSP) and determined the time of the peak. The amplitude of each evoked EPSP (or IPSP) on single trials was taken as the difference between the postsynaptic *V*_m_ at the peak time of the average EPSP (or IPSP) after the AP and the *V*_m_ before onset of the current pulse evoking the AP. We measured the baseline activity as the difference in *V*_m_ over the same time delay, but without a presynaptic AP. The rise time of the monosynaptic IPSP was measured as the time from 20% to 80% of the peak amplitude, and the decay time constant was obtained through a single exponential fit to the decay phase.

## Supporting Information

Figure S1 A linear relationship between amplitudes of disynaptic IPSPs at depolarized and resting *V*_m_. Dashed line is the line of y = x. Red line, a linear regression fit (y = x+0.53) with the slope fixed at 1. This linear regression function was predicted by the hyperbolic function (y = 100%+0.67/x) that fits the data shown in Figure 1F well.
(TIF)

Figure S2 Identification of PC, LTS, and FS cells. (A) An example image (DAB staining) of a PC-LTS pair. The LTS cell was indicated by the arrowhead. Scale bar: 10 µm. (B) Distinct firing patterns of the PC and the LTS cell. See also the Methods. (C) PC depolarization enhanced the summated EPSPs (evoked by AP burst at the presynaptic PC) at the LTS cell. (D) Averaged EPSPs at the depolarized *V*_m_ (red) were larger than those at resting *V*_m_ of the PC. Note that the LTS cell received facilitating EPSPs. Panels A–D, same pair. (E) An example image of a PC-FS pair. The FS cell was indicated by the arrowhead. Scale bar: 10 µm. (F) Firing pattern of the FS cell. (G) PC depolarization significantly increased the average amplitude of the EPSPs evoked by single APs (*p*<0.001). Same protocol as shown in Figure 6A. Inset, depressing EPSPs recorded at the FS cell in response to an AP burst at the presynaptic PC. Panels E–G, same pair.
(TIF)

Figure S3 Measurements for peak amplitude of individual EPSPs during an AP train. Top, a train of APs was evoked through current injection in the presynaptic PC; bottom, facilitating EPSPs recorded at an LTS interneuron. The peak amplitude of individual EPSPs was obtained by measuring the voltage difference between the peak of an EPSP and the baseline *V*_m_. The peak amplitudes were then normalized to the 6^th^ EPSP (while presynaptic *V*_m_ was at resting) and averaged (as shown in Figure 4E).
(TIF)

Figure S4 PC depolarization increases the number and decreases the onset latency of LTS APs. (A) Left, overlay of example postsynaptic responses of LTS to a train of presynaptic APs (10 APs fired at a relatively low frequency of 20 Hz) at resting presynaptic *V*_m_ (−65 mV). Right, rasters and peristimulus histogram showing the number and timing of APs in LTS across trials. (B) Same cell as in (A). Presynaptic *V*_m_ was depolarized to −42 mV. Notice the increase in number of APs and the decrease in AP onset latency.
(TIF)

Figure S5 *V*_m_-dependent modulation of recurrent inhibition was dependent of the inhibition of D-current. (A) Example recording from a PC-PC pair that had reciprocal disynaptic IPSPs. (B) Same recording as shown in (A). In control condition, the size of disynaptic IPSPs was larger after presynaptic depolarization (red) in comparison with that at resting *V*_m_. Blocking D-current with bath application of a low concentration of 4-AP (50 µM) increased the amplitude and the integrated voltage area and shortened the onset latency of disynaptic IPSPs, but no additional changes were observed after presynaptic depolarization. Arrows indicate the onset of stimulation. (C) Group data (*n* = 7 connections in 6 PC-PC pairs) showing that 4-AP abolished the presynaptic depolarization-induced facilitation of disynaptic IPSPs. ** *p*<0.01.
(TIF)

## Abbreviations

AP: action potential
EPSP: excitatory postsynaptic potential
FS: fast spiking interneuron
LTS: low-threshold spiking interneuron
PC: pyramidal cell
RMP: resting membrane potential
*V*_m_: membrane potential
