# Supplementary figures and images for: Membrane Potential-Dependent Modulation of Recurrent Inhibition in Rat Neocortex
Source: PLoS Biol. 2011 Mar 22;9(3):e1001032. doi: 10.1371/journal.pbio.1001032 (PMC3062529; doi:10.1371/journal.pbio.1001032)

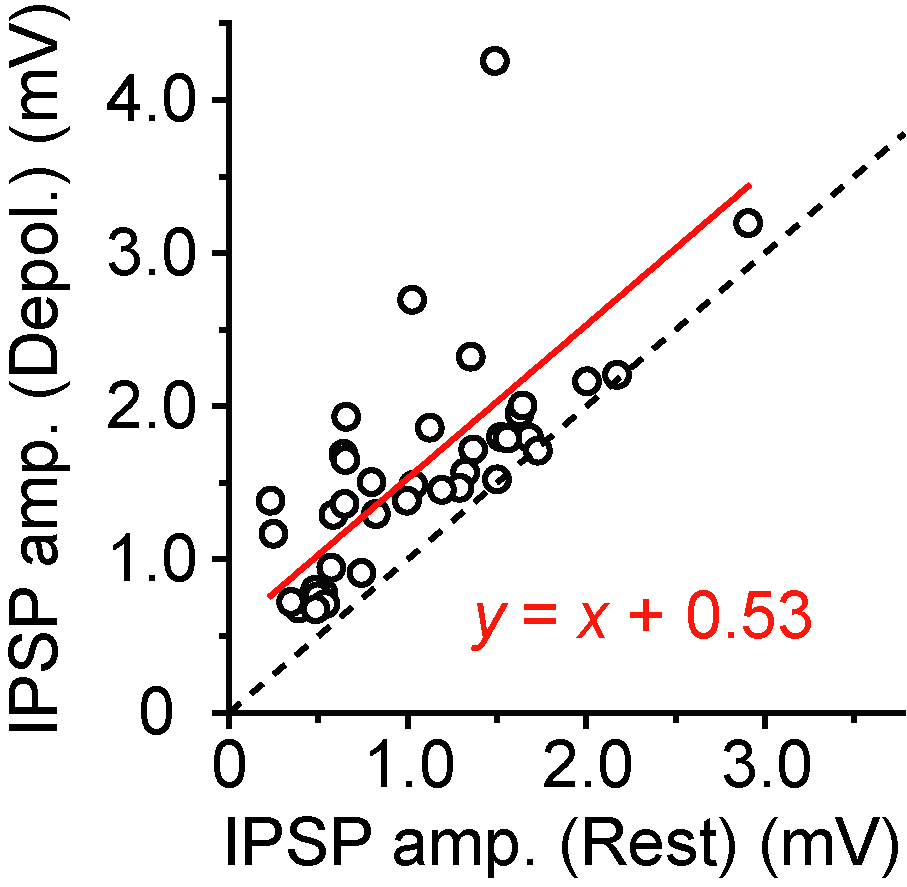

Supplement: Figure S1 — A linear relationship between amplitudes of disynaptic IPSPs at depolarized and resting V m. Dashed line is the line of y = x. Red line, a linear regression fit (y = x+0.53) with the slope fixed at 1. This linear regression function was predicted by the hyperbolic function (y = 100%+0.67/x) that fits the data shown in Figure 1F well. (TIF) [file pbio.1001032.s001.tif]

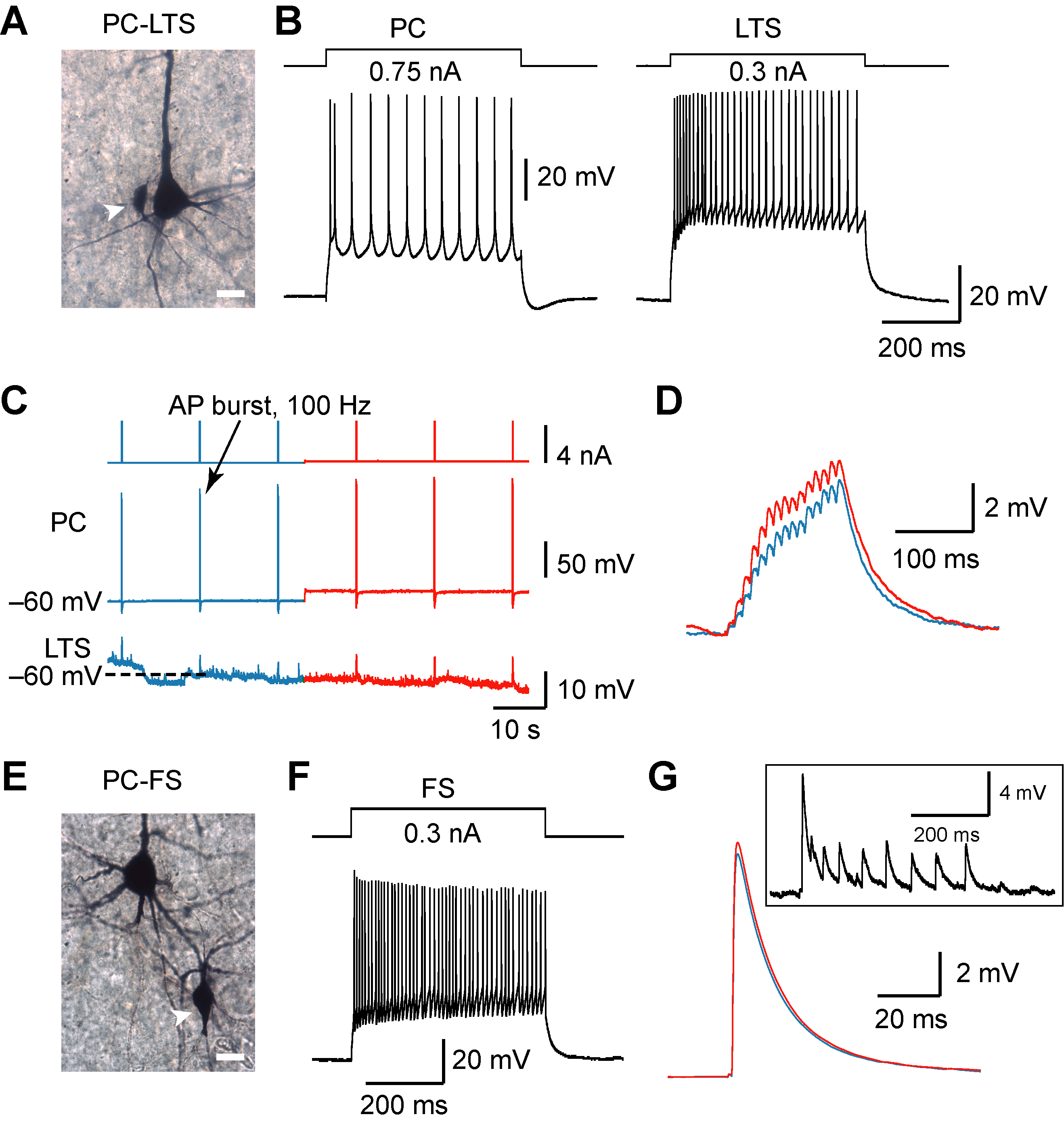

Supplement: Figure S2 — Identification of PC, LTS, and FS cells. (A) An example image (DAB staining) of a PC-LTS pair. The LTS cell was indicated by the arrowhead. Scale bar: 10 µm. (B) Distinct firing patterns of the PC and the LTS cell. See also the Methods. (C) PC depolarization enhanced the summated EPSPs (evoked by AP burst at the presynaptic PC) at the LTS cell. (D) Averaged EPSPs at the depolarized V m (red) were larger than those at resting V m of the PC. Note that the LTS cell received facilitating EPSPs. Panels A–D, same pair. (E) An example image of a PC-FS pair. The FS cell was indicated by the arrowhead. Scale bar: 10 µm. (F) Firing pattern of the FS cell. (G) PC depolarization significantly increased the average amplitude of the EPSPs evoked by single APs (p<0.001). Same protocol as shown in Figure 6A. Inset, depressing EPSPs recorded at the FS cell in response to an AP burst at the presynaptic PC. Panels E–G, same pair. (TIF) [file pbio.1001032.s002.tif]

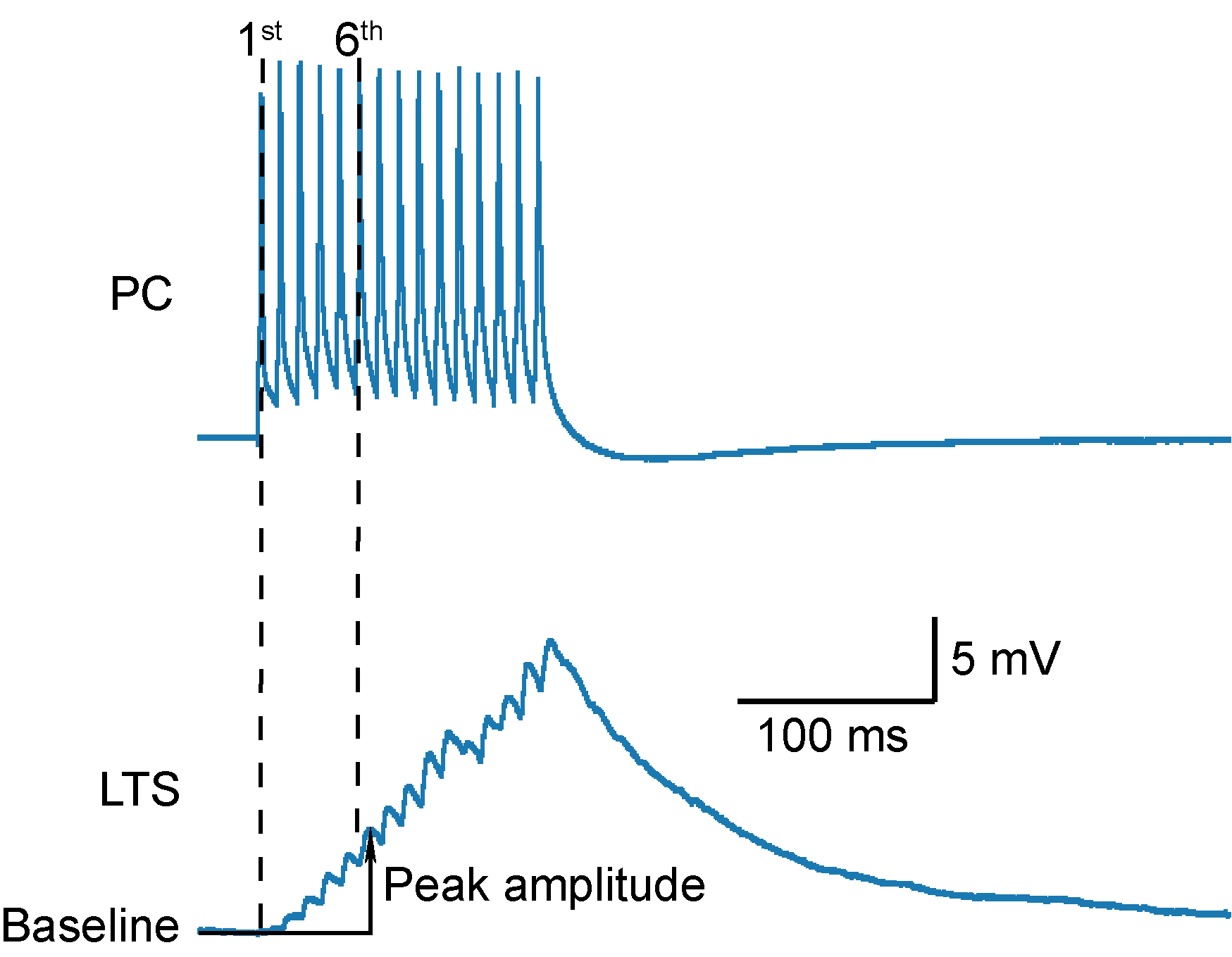

Supplement: Figure S3 — Measurements for peak amplitude of individual EPSPs during an AP train. Top, a train of APs was evoked through current injection in the presynaptic PC; bottom, facilitating EPSPs recorded at an LTS interneuron. The peak amplitude of individual EPSPs was obtained by measuring the voltage difference between the peak of an EPSP and the baseline V m. The peak amplitudes were then normalized to the 6th EPSP (while presynaptic V m was at resting) and averaged (as shown in Figure 4E). (TIF) [file pbio.1001032.s003.tif]

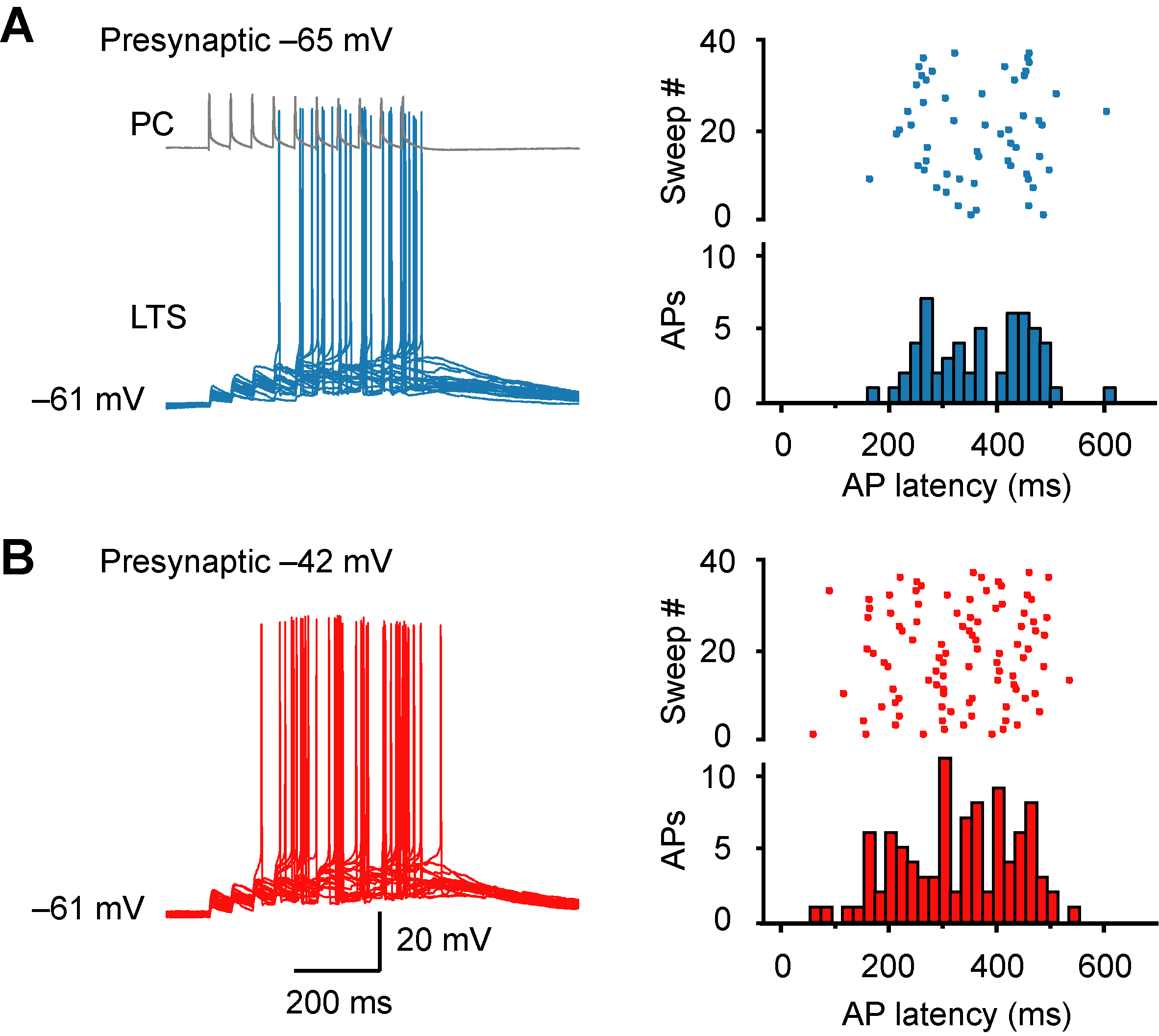

Supplement: Figure S4 — PC depolarization increases the number and decreases the onset latency of LTS APs. (A) Left, overlay of example postsynaptic responses of LTS to a train of presynaptic APs (10 APs fired at a relatively low frequency of 20 Hz) at resting presynaptic V m (−65 mV). Right, rasters and peristimulus histogram showing the number and timing of APs in LTS across trials. (B) Same cell as in (A). Presynaptic V m was depolarized to −42 mV. Notice the increase in number of APs and the decrease in AP onset latency. (TIF) [file pbio.1001032.s004.tif]

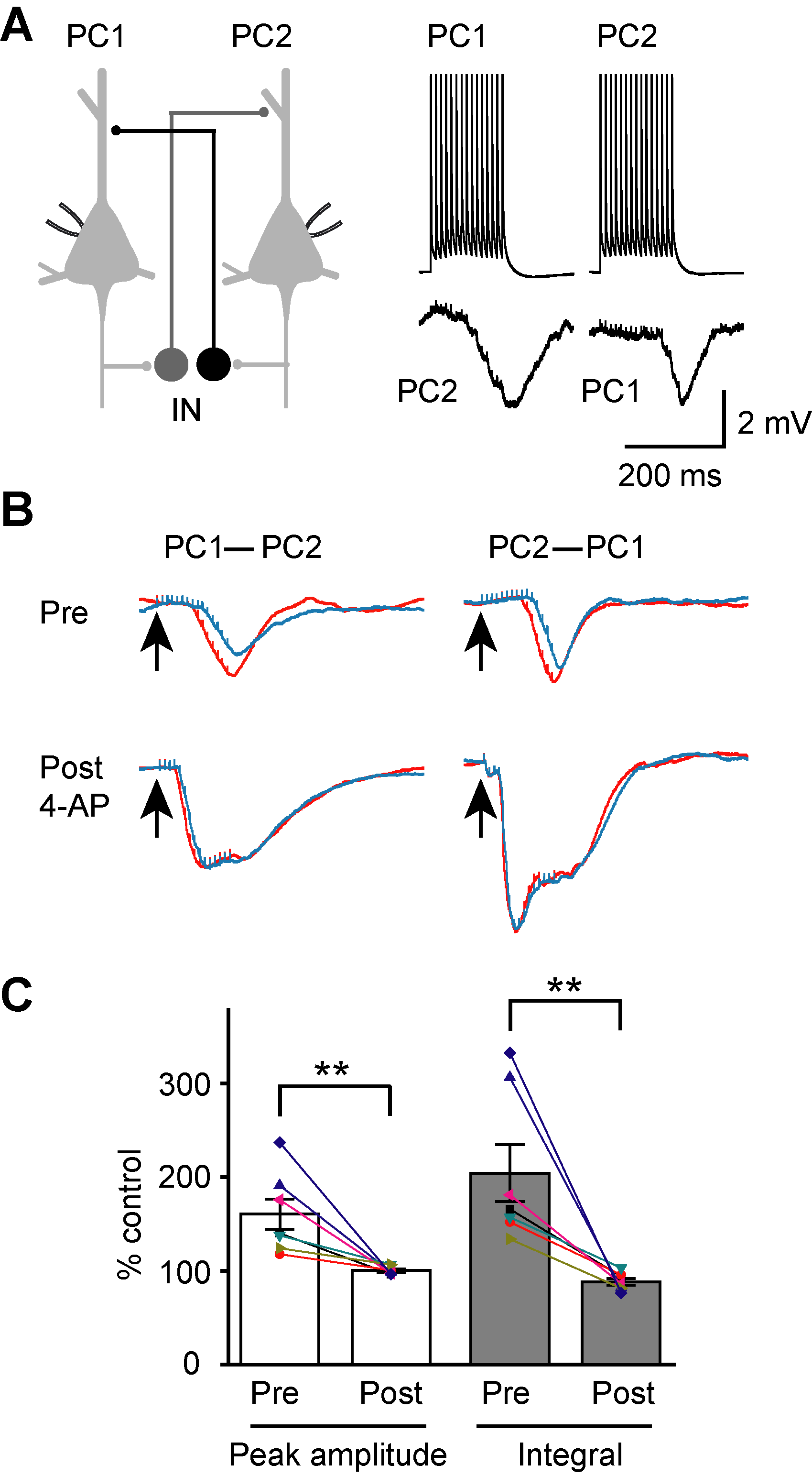

Supplement: Figure S5 — V m-dependent modulation of recurrent inhibition was dependent of the inhibition of D-current. (A) Example recording from a PC-PC pair that had reciprocal disynaptic IPSPs. (B) Same recording as shown in (A). In control condition, the size of disynaptic IPSPs was larger after presynaptic depolarization (red) in comparison with that at resting V m. Blocking D-current with bath application of a low concentration of 4-AP (50 µM) increased the amplitude and the integrated voltage area and shortened the onset latency of disynaptic IPSPs, but no additional changes were observed after presynaptic depolarization. Arrows indicate the onset of stimulation. (C) Group data (n = 7 connections in 6 PC-PC pairs) showing that 4-AP abolished the presynaptic depolarization-induced facilitation of disynaptic IPSPs. ** p<0.01. (TIF) [file pbio.1001032.s005.tif]
